# Dual Role of Transcription Factors in the Development and Thermogenic Function of Brown Adipose Tissue

**DOI:** 10.7150/ijms.127214

**Published:** 2026-02-04

**Authors:** Shuai Wang, Fuan Xie, Weihua Li, Wenlong Xie, Hongqiu Cheng

**Affiliations:** 1 Department of Hepatology and Infectious Diseases, the Second Affiliated Hospital of Shantou University Medical College, Shantou, Guangdong, China.; 2 School of Basic Medical Sciences, Yichun University, Yichun 336000, China.; 3 Xiamen Treatgut Biotechnology Co., Ltd., Xiamen, Fujian 361101, China.; 4 Department of Cardiology, Xiamen Key Laboratory of Cardiac Electrophysiology, Xiamen Institute of Cardiovascular Diseases, The First Affiliated Hospital of Xiamen University, School of Medicine,Xiamen University, Xiamen 361003, China.

**Keywords:** brown adipose tissue, transcription factors, development, thermogenesis

## Abstract

Obesity, a major global health challenge, is intricately linked to various metabolic disorders, primarily driven by an imbalance between energy intake and expenditure. Brown adipose tissue (BAT), through its unique thermogenic capability, dissipates energy as heat, playing a vital role in regulating energy homeostasis and maintaining body temperature. Recent studies have revealed a complex regulatory network involving multiple transcription factors (e.g., PPARγ, EBF2, ZFP516, FOXP1) and signaling pathways (e.g., cAMP-PKA, AMPK, mTOR), which act synergistically to finely tune the development and thermogenic function of BAT. Furthermore, gene therapy based on adeno-associated virus (AAV) vectors, which enhances the thermogenic capacity of BAT, provides a highly promising strategy for addressing obesity and metabolic disorders. Additionally, several natural product extracts including *Artemisia argyi* oil and capsaicin may activate BAT thermogenesis with fewer side effects, representing potential safe and effective dietary supplements for combating obesity in the future. A deeper understanding of these mechanisms may lead to novel therapeutic approaches targeting obesity and metabolic disorders, paving the way for new interventions to improve human health.

## Introduction

Obesity is a complex metabolic disorder that has reached epidemic proportions worldwide^1^. It is strongly linked to various severe health conditions, such as insulin resistance, type 2 diabetes, cardiovascular diseases, and certain types of cancer^2^, ^3^. The root cause of obesity lies in an imbalance between energy intake and expenditure, leading to the excessive expansion of adipose tissue^4^-^6^. This expansion primarily occurs in adipose tissue, where both hypertrophy (increased cell size) and hyperplasia (increased cell number) contribute to the accumulation of fat^7^, ^8^. When the capacity of adipose tissue to store excess energy is exceeded, free fatty acids (FFAs) accumulate, contributing to lipotoxicity and the development of metabolic disorders^9^. Adipose tissue, a key player in energy balance regulation, has traditionally been categorized into three types: white adipose tissue (WAT), brown adipose tissue (BAT), and beige adipose tissue^10^, ^11^. WAT primarily serves as a reservoir for triglycerides, mobilizing fatty acids during energy expenditure^12^. Beige adipose cells originate from precursor cells expressing platelet-derived growth factor receptor-α (Pdgfα^+^) and stem cell antigen 1 (Sca1^+^), as well as from myoglobin heavy chain 11 positive (Myh11^+^) progenitor cells^13^. Besides the de novo differentiation from these precursor cells, research has indicated that beige adipose cells can also transdifferentiate directly from mature white adipocytes^14^. Under cold stimulation, beige adipose tissues engage in non-shivering thermogenesis, utilizing glucose and triglycerides (TG) to maintain body temperature homeostasis^15^. Due to their ability to increase energy expenditure, beige adipocytes have emerged as a promising target for addressing obesity and enhancing metabolic profiles^16^. Similar to beige adipose tissue, BAT is specialized in thermogenesis, a process by which energy is dissipated as heat to maintain body temperature^17^, ^18^. In newborn humans, BAT is predominantly distributed in the interscapular, cervical, axillary, and perirenal regions, where it serves a critical role in body temperature homeostasis via non-shivering thermogenesis^19^. In adults, BAT localizes primarily to the supraclavicular and cervical regions, with metabolically active BAT correlating with reduced body weight^20^. Therefore, promoting the development and function of BAT could be a potential strategy for combating obesity and its associated metabolic disorders.

Lineage-tracing studies in mice have provided insight into the developmental origins of brown adipocytes, demonstrating that BAT, along with skeletal muscle, dorsal dermis, and certain subpopulations of white adipocytes, derives from the dermomyotome (DM) region of somites^21^, ^22^. This lineage is characterized by the expression of key transcription factors, including *Myf5*, *En1*, and *Pax7*^23^-^25^. Among the signaling pathways that regulate the differentiation of brown adipocytes, bone morphogenetic protein 7 (BMP7) has emerged as a crucial mediator^26^. BMP7 promotes the commitment of brown preadipocytes by inducing the expression of PR domain containing 16 (PRDM16), a master regulator of brown fat differentiation that also orchestrates the balance between brown adipocytes and skeletal muscle in *Myf5^+^* progenitors^27^, ^28^. Moreover, BMP7 activates peroxisome proliferator-activated receptor gamma coactivator 1α (PGC-1α), which further stimulates the expression of thermogenic markers such as uncoupling protein 1 (UCP1) and enhances mitochondrial biogenesis^27^, ^29^, ^30^. Thermogenic gene expression in brown adipocytes is additionally regulated by the p38 mitogen-activated protein kinase (MAPK) signaling pathway, which is activated by β-adrenergic agonists essential for thermogenic activation^31^, ^32^. The p38 MAPK cascade phosphorylates key transcription factors, including activating transcription factor 2 (ATF2) and PGC-1α, leading to the upregulation of *Ucp1* and other mitochondrial genes that drive heat production^33^-^35^.

This review summarizes recent insights into the transcription factors that regulate both the development and thermogenic function of BAT, emphasizing the complex interplay between transcriptional regulators and signaling pathways. Key transcription factors, including Peroxisome Proliferator-Activated Receptor Gamma (PPARγ), CCAAT/Enhancer Binding Protein Beta (C/EBPβ), PGC-1α, and PRDM16, are essential for initiating and maintaining BAT-specific gene programs. Other factors such as EBF2, ZFP516, and FOXP1 are crucial for lineage commitment, thermogenic gene expression, and metabolic regulation. Additionally, SIRT family members contribute to energy homeostasis through deacetylation and regulation of mitochondrial biogenesis. The Kruppel-like factor family, along with transcription factors such as DDB1, ATF4, and OVOL2, further modulates BAT development and thermogenesis by influencing gene expression and chromatin accessibility. Major signaling pathways, including cAMP-PKA, AMPK, mTOR, and TGF-β/BMP, interact with these transcription factors to finely tune the thermogenic response. The review also explores the potential of gene therapy using AAV vectors to enhance BAT function, proposing new therapeutic strategies for combating obesity and metabolic disorders by targeting the intricate regulatory network governing BAT.

## The previous major transcriptional elements for BAT development and thermogenesis

A large number of transcription factors have been identified that regulate the development and thermogenesis of BAT, both positively and negatively (**Table 1**). Notably, many of these regulatory factors operate through four key transcription elements: PPARγ, C/EBPβ, PGC-1α, and PRDM16^36^. PPARγ plays a critical role in BAT development and the initiation of thermogenic programs^37^. Mice lacking PPARγ exhibit a morphological “whitening” of BAT, highlighting its essential function in brown adipocyte development^38^, ^39^. Notably, BAT from C/EBPβ-knockout mice exhibits a widespread reduction in BAT-selective gene expression, accompanied by a significant increase in the expression of skeletal muscle genes^40^. PPARγ and C/EBPβ function as transcription factors that directly bind to DNA^40^. Similar to the phenotype observed in C/EBPβ-knockout mice, the loss of PRDM16 from brown fat precursors results in the loss of brown fat characteristics and promotes muscle differentiation^41^. Indeed, PRDM16 functions as a transcriptional co-regulator that forms a complex with canonical DNA-binding transcription factors such as C/EBPβ via its zinc finger domains, which specifically governs the bidirectional cell fate switch between skeletal myoblasts and brown adipocytes^42^, ^43^. Mitochondria are essential for maintaining the thermogenic functions of BAT and regulating energy expenditure, with UCP-1 serving as a critical component located in the inner mitochondrial membrane, facilitating these processes^44^. PGC-1α serves as a master regulator of mitochondrial biogenesis, with its loss leading to reduced mitochondrial density and UCP-1 protein levels, which contributes to cold sensitivity and obesity^45^. Maternal exposure to glucocorticoids during pregnancy enhances DNA methylation in the promoter region of *Pgc-1α*, which impairs mitochondrial biogenesis and predisposes offspring to metabolic dysfunction^46^.

### Early B cell factor-2

**Figure 1:**
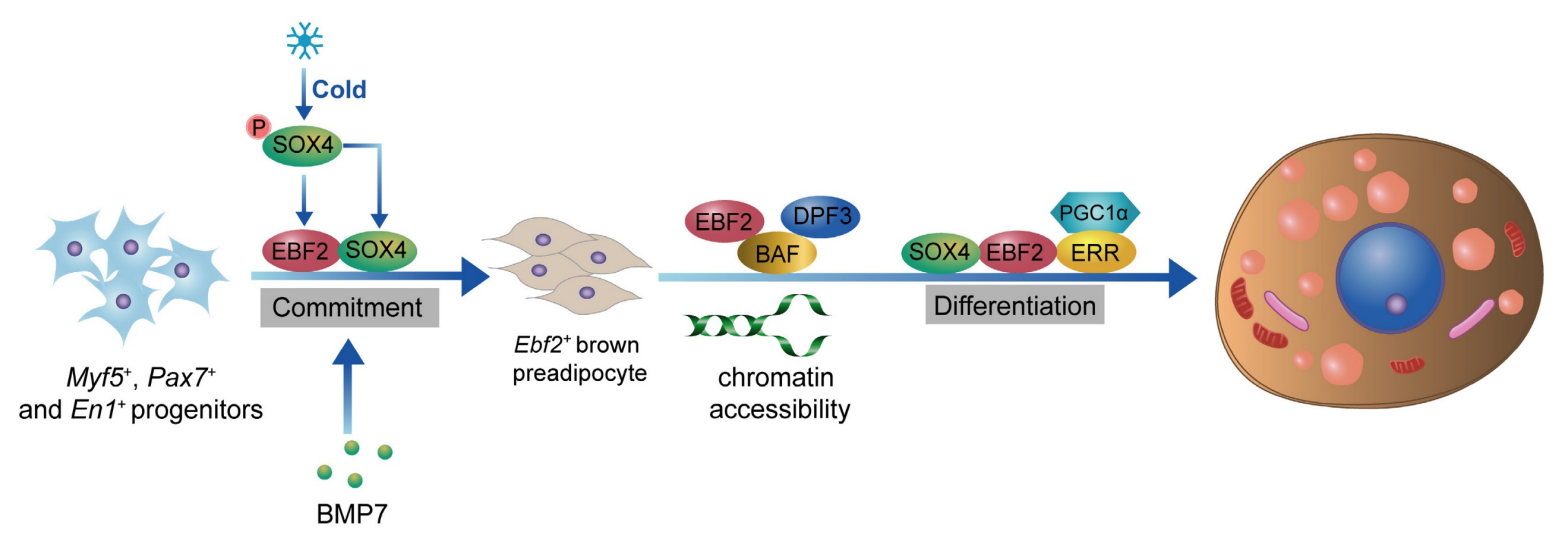
** Role of EBF2 in BAT development and thermogenesis**. EBF2 is crucial for the development of BAT, as it regulates the molecular profile of adipogenic precursor cells committed to the brown adipocyte lineage. The activation of Ebf2 by SOX4 further facilitates BAT development and thermogenesis. In mature brown adipocytes, EBF2 maintains the expression of key thermogenic genes, including* Ucp1* and *Prdm16*, which dissipates energy as heat. EBF2 cooperates with transcription factors like PPARγ and interacts with the chromatin remodeling complex BAF and histone reader DPF3 to enhance chromatin accessibility at BAT-specific enhancers. Additionally, EBF2 enhances the activity of the ERRα/PGC1α complex, promoting thermogenic gene expression and thermogenesis.

EBF2 is a critical transcription factor that plays a pivotal role in the development and thermogenic function of BAT^47^, ^48^. The loss of Ebf2 leads to impaired BAT development, reduced thermogenic gene expression, and increases susceptibility to obesity and metabolic disorders^48^, ^49^. EBF2 is selectively expressed in brown and beige adipogenic precursor cells during embryogenesis and adulthood^47^. EBF2 marks and regulates the molecular profile of these precursor cells committed to the brown adipocyte lineage. EBF2 also recruits PPARγ to brown fat-specific gene targets, including *Prdm16* and *Ucp1*, to maintain brown adipocyte identity and reprogram cells towards a brown fat fate^48^. Recent studies indicate that GATA6, acting upstream of EBF2, binds to the promoter region of *Ebf2*, thereby enhancing the expression of Ebf2 and playing a pivotal role in the fate determination of brown adipocyte progenitors^50^. In mature brown adipocytes, EBF2 is essential for maintaining the expression of key thermogenic genes such as *Prdm16* and* Ucp1*^51^. EBF2 cooperates with other transcription factors like PPARγ to activate the expression of thermogenic genes, thereby enhancing the thermogenic capacity of brown adipocytes^52^. Additionally, EBF2 interacts with the chromatin remodeling complex BAF and its histone reader subunit DPF3 to regulate chromatin accessibility at brown fat-specific enhancers, facilitating the transcription of thermogenic genes^49^. EBF2 also enhances the transcriptional activity of the ERRα/PGC-1α complex, promoting the expression of thermogenic genes and maintaining core body temperature and thermogenic function in BAT^51^. Recent studies on SRY-related High Mobility Group box transcription factor 4 (SOX4) have further clarified its role in driving BAT development and thermogenesis by activating EBF2 transcription^53^. Additionally, the complex formed by SOX4 and EBF2 synergistically facilitates both the development and thermogenesis of BAT^53^. Besides activating EBF2 transcription, SOX4 also forms distinct complexes with EBF2 and PPARγ, respectively, to synergistically enhance the expression of thermogenic genes in mature brown adipocytes^54^. Interestingly, overexpression of Ebf2 in C2C12 myoblasts significantly reduced muscle-specific genes like *MyoD* and *MyoG*, indicating that Ebf2 reprograms myoblasts towards a brown-preadipose-like state, redirecting them away from muscle differentiation^47^. Therefore, EBF2 acts as a central regulator of BAT development and thermogenesis, positioning it as a potential therapeutic target for combating obesity and related metabolic disorders (**Figure 1**).

### Zinc finger protein 516

**Figure 2:**
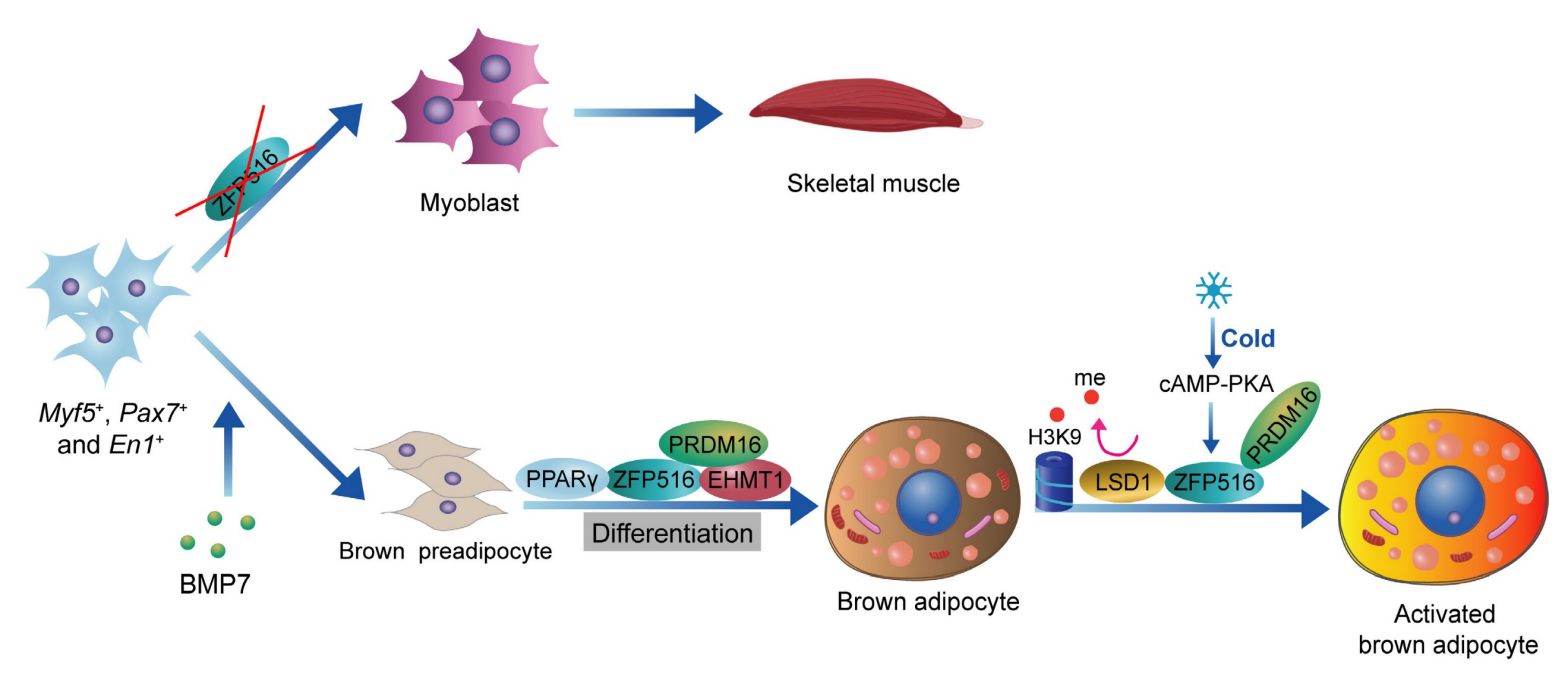
** Role of ZFP516 in BAT development and thermogenesis.** ZFP516 promotes brown adipogenesis and regulates BAT-specific gene expression through interaction with the EHMT1-PRDM16 complex. In mature brown adipocytes, ZFP516 binds directly to the Ucp1 promoter to drive its transcriptional activation. Cold-induced β-adrenergic signaling upregulates ZFP516 expression, further boosting thermogenic gene activity.

ZFP516 is a key transcription factor in the development and thermogenic function of BAT^55^. During BAT development, ablation of Zfp516 blocks brown fat development and shows drastically reduced BAT mass, while promoting myogenesis^56^. Ablation of Zfp516 impairs adipogenesis of BAT, while concurrently upregulating the expression of muscle-specific genes, including *MyoD*, *MyoG*, *Mck*, *Myf5*, and *Myf6*^56^. Moreover, ectopic expression of Zfp516 was sufficient to reprogram C2C12 myoblasts into brown adipocytes^56^. ZFP516 interacts with the EHMT1-PRDM16 complex to form a transcriptional complex that drives the expression of BAT-selective genes^57^, ^58^. In mature brown adipocytes, cold stimulation activates the cAMP-CREB/ATF2 signaling pathway via β-adrenergic receptors, which induces the expression of ZFP516^42^, ^56^. ZFP516, acting as a transcription factor, binds to the CCACT sequence within the *Ucp1* promoter region (from -70 to -45 bp) via its N-terminal domain, activating transcription of *Ucp1*. In addition, ZFP516 also binds to the -2.4 kb region of the *Pgc-1α* promoter and the -2.8 kb region of the *Cox8b* promoter, enhancing their transcriptional activity^18^, ^56^. Recent studies indicate that lysine-specific demethylase 1 (LSD1) is a direct binding partner of Zfp516. Through its interaction with Zfp516, LSD1 is recruited to the promoter regions of *Ucp1* and other BAT-enriched genes, such as *Pgc-1α*, where it functions as a co-activator by demethylating H3K9^59^. Overexpression of Zfp516 in adipose tissue promotes the browning of iWAT even at room temperature, resulting in increased body temperature and energy expenditure, while preventing diet-induced obesity^56^. ZFP516 is a key regulator of BAT development and thermogenesis, promoting the browning of iWAT and enhancing energy expenditure, making it a promising target for anti-obesity therapies^60^ (**Figure 2**).

### Forkhead box protein P1

**Figure 3:**
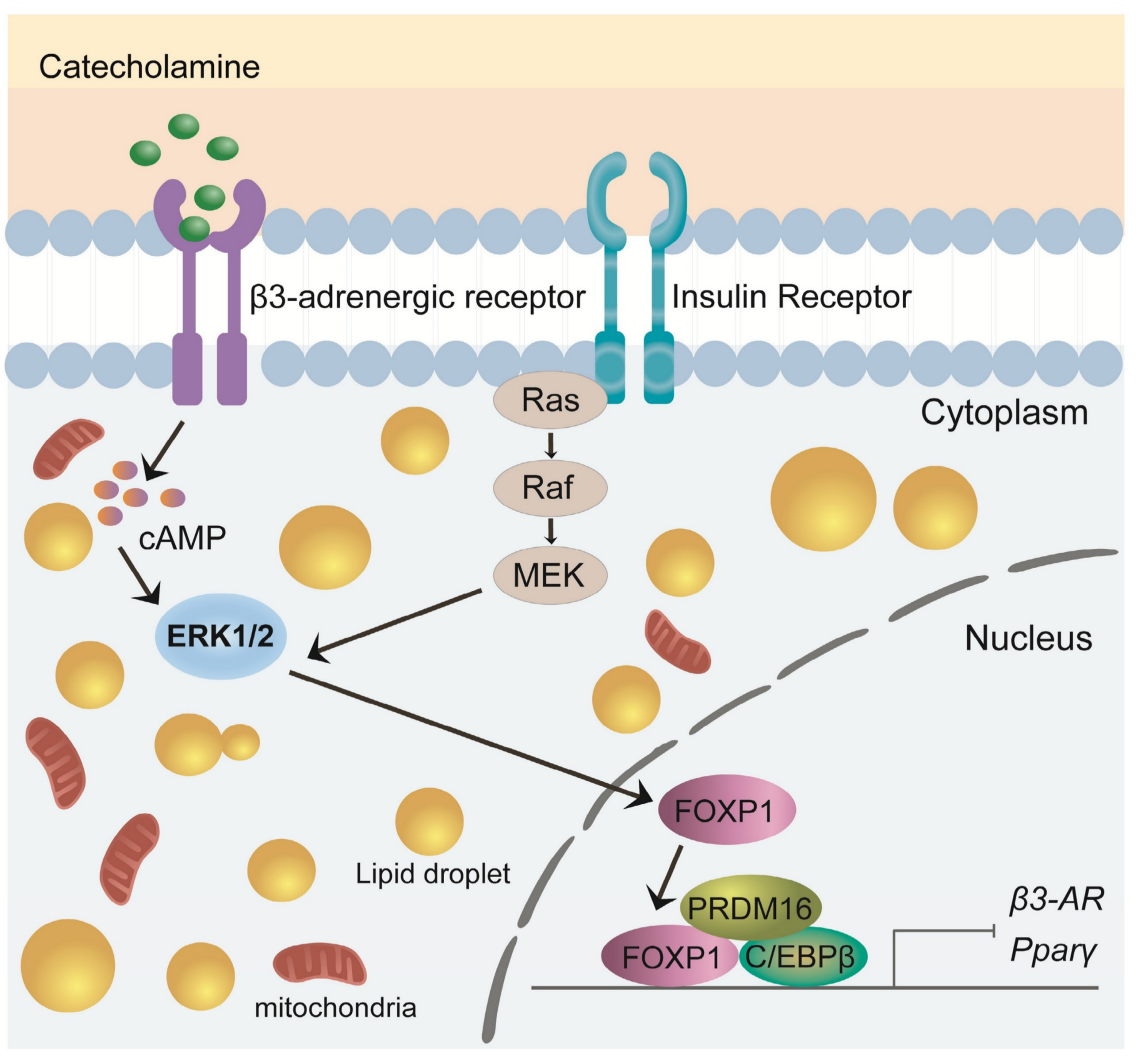
** Role of FOXP1 in BAT development and thermogenesis.** Foxp1 overexpression disrupts thermogenesis and increases susceptibility to diet-induced obesity. FOXP1 regulates β3-adrenergic receptor expression via the β3-AR/cAMP/ERK1/2 signaling pathway and forms complexes with Prdm16 and C/EBPβ to inhibit PPARγ and β3-AR expression.

FOXP1 is a critical transcription factor involved in cell differentiation, development, and metabolic regulation^61^. Recent studies have highlighted its pivotal role in brown adipocyte development and thermogenesis, particularly in modulating the expression and function of the β3-adrenergic receptor (β3-AR)^55^, ^62^. Foxp1 depletion in BAT progenitors enhances brown adipocyte differentiation and promotes the expression of key BAT-selective genes (*Ucp1*, *Dio2*, *Prdm16*) and mitochondrial genes (*Cox7a1*, *Cox8b*, *Cox5b*). In contrast, Foxp1 overexpression disrupts adaptive thermogenesis and predisposes mice to diet-induced obesity^61^. Upon cold exposure, the sympathetic nervous system (SNS) releases adrenergic signals, such as noradrenaline, which activate the β3-AR/cAMP/ERK1/2 signaling pathway in BAT^63^-^65^. The expression of Foxp1 in stromal vascular fraction (SVF) cells of BAT is regulated by the β3-AR/ERK1/2 pathway^61^, ^65^. Conversely, Foxp1 directly represses β3-AR transcription in adipocytes. Furthermore, FOXP1 forms a complex with PRDM16 and C/EBPβ proteins to inhibit the expression of PPARγ and β3-AR^61^. These findings highlight FOXP1 as a key regulator of development and thermogenesis in BAT, offering valuable insights for developing potential therapeutic strategies targeting obesity and metabolic disorders (**Figure 3**).

### SIRT family members

The sirtuins are a family of highly conserved NAD^+^-dependent enzymes, with mammals possessing seven sirtuins, named SIRT1-7^66^. Members of the SIRT family not only exhibit deacetylase activity but also function as transcription factors, playing a critical role in the regulation of adipocyte differentiation and thermogenesis^67^, ^68^.

**Figure 4:**
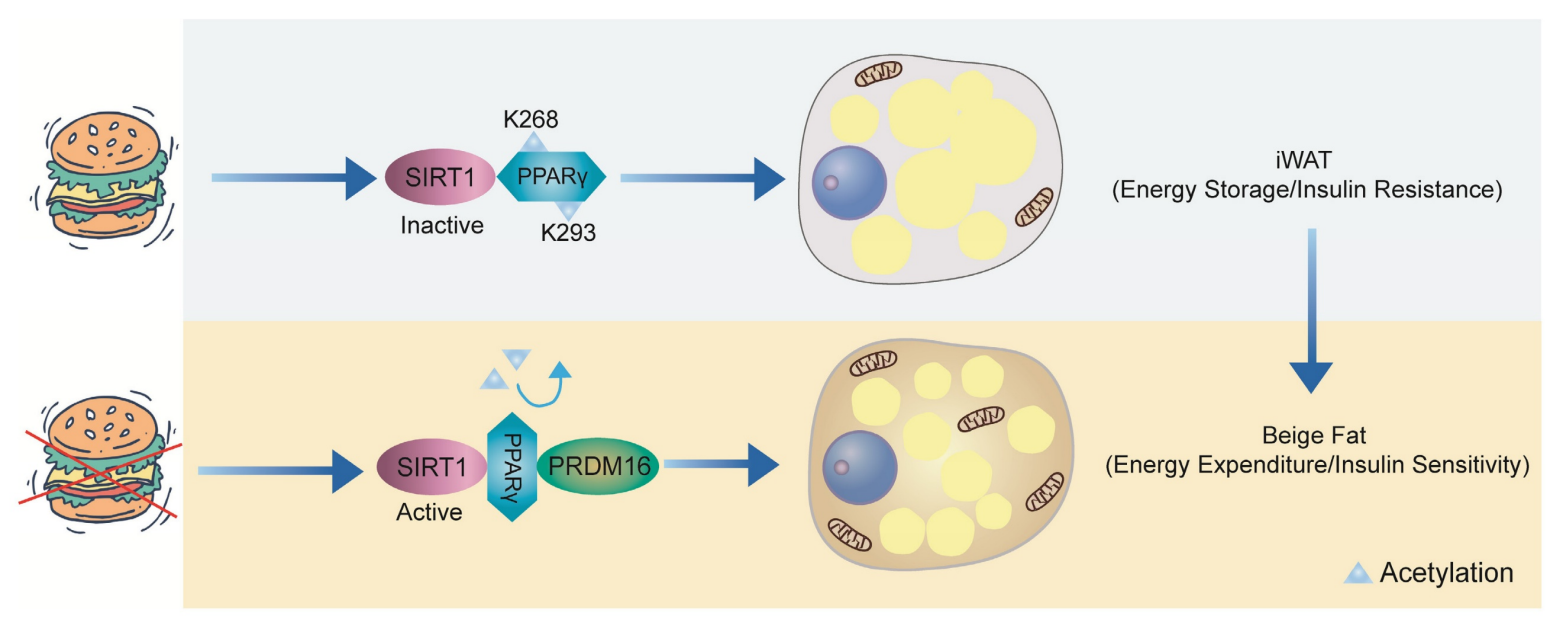
** SIRT1-mediated deacetylation of PPARγ in WAT**. When there is an abundance of nutrients, SIRT1 remains inactive, leading to the acetylation of PPARγ at Lys268 and Lys293, which favors lipid accumulation. However, during energy deprivation, SIRT1 becomes activated and is recruited to PPARγ, likely due to ligand-induced conformational changes, where it deacetylates Lys268 and Lys293. In WAT, the deacetylation of PPARγ enhances its interaction with PRDM16, thereby promoting thermogenesis and improving insulin sensitivity.

SIRT1, an NAD⁺-dependent deacetylase, plays a crucial role in maintaining energy homeostasis in adipose tissue^69^, ^70^. When nutrients are abundant, SIRT1 remains inactive, and PPARγ is acetylated at Lys268 and Lys293, promoting lipid storage. However, during energy deprivation, SIRT1 becomes activated and is recruited to PPARγ, likely due to ligand-induced conformational changes, where it deacetylates Lys268 and Lys293. In WAT, the deacetylation of PPARγ enhances its interaction with PRDM16, thereby promoting thermogenesis (energy expenditure) and improving insulin sensitivity^71^, ^72^ (**Figure 4**).

**Figure 5:**
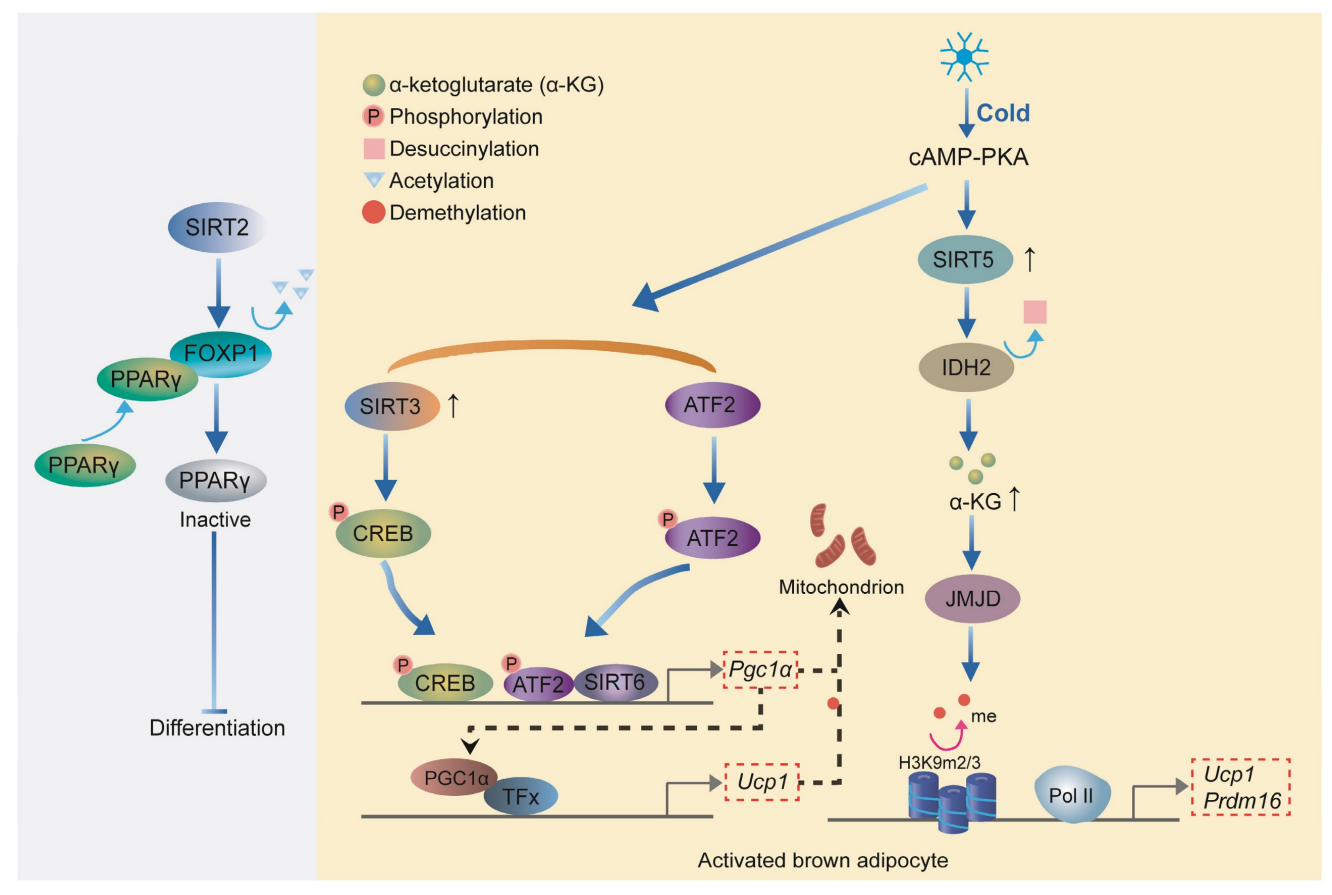
** Role of Sirtuins in adipocyte differentiation and thermogenesis**. SIRT2 inhibits adipogenesis by deacetylating FOXO1, which represses PPARγ activity. SIRT3 enhances thermogenesis in brown adipocytes by activating CREB and upregulating PGC-1α. SIRT6 promotes thermogenesis by facilitating phospho-ATF2 binding to the *Pgc-1α* promoter, enhancing *Ucp1* expression. SIRT5 supports brown adipocyte differentiation through the de-succinylation of IDH2, increasing α-KG levels and promoting PPARγ and PRDM16 expression.

Other transcription factors in the SIRT family also play an important role in the development and thermogenesis of BAT. SIRT2 inhibits adipogenesis by deacetylating Forkhead Box Protein O1 (FOXO1), which enhances FOXO1 binding to PPARγ and subsequently represses PPARγ transcriptional activity^73^, ^74^. Upon cold exposure, SIRT3 enhances thermogenesis in brown adipocytes by stimulating CREB phosphorylation, which directly activates the *Pgc-1α* promoter, thereby upregulating key thermogenic genes such as *Ucp1*^75^. Overexpression of SIRT6 in brown adipocytes stimulates the thermogenic program. SIRT6 interacts with and enhances phospho-ATF2 binding to the PGC-1α gene promoter, thereby activating its expression^68^. PGC-1α, as a transcriptional cofactor, binds to the promoter region of *Ucp1* along with other transcription factors, promoting the expression of Ucp1 and other thermogenic genes^76^. Loss of SIRT5 significantly impairs brown adipocyte differentiation, as demonstrated by reduced lipid accumulation, compromised cellular respiration, and downregulation of key adipogenic genes, including *Pparγ*, *Prdm16*, and *Ucp1*^77^. SIRT5 promotes the de-succinylation of isocitrate dehydrogenase 2 (IDH2), increasing intracellular α-ketoglutarate (α-KG) levels, which in turn activates histone demethylases^77^. These demethylases remove H3K9me2 and H3K9me3 marks from the promoters of *Pparγ* and *Prdm16*, thereby enhancing their expression to promote brown adipocyte differentiation and thermogenesis^78^. Collectively, these findings highlight the critical roles of sirtuins in regulating adipocyte differentiation and thermogenesis, with each member contributing through distinct mechanisms to maintain energy homeostasis (**Figure 5**).

### Kruppel-like factor family

Members of the KLF family are transcription factors with zinc finger domains that bind to specific DNA sequences, such as GC-rich and CACCC-box motifs^79^, ^80^. The KLF family plays a crucial role in the development and thermogenesis of BAT (Table 2). KLF2, KLF3, and KLF7 inhibit brown adipocyte differentiation and thermogenesis by suppressing the expression of C/EBPα and PPARγ^81^, ^82^. KLF6 directly binds to the promoter regions of Pparγ and C/ebpα, activating their expression^83^, ^84^. The expression of C/EBPβ and C/EBPδ can subsequently activate the expression of the *Klf5* gene^85^, ^86^. KLF5 and KLF15 further enhance the expression of PPARγ2, ultimately promoting adipocyte differentiation^81^, ^87^. KLF9 is induced under cold exposure and promotes the expression of Pgc-1α^88^. KLF11 significantly increases Ucp1 expression by binding to the GC box (CGCCC or GCGGG) in the *Ucp1* promoter^89^. KLF15 enhances UCP1 expression by binding to the GT boxes (CACCC or GTGGG) in the *Ucp1* promoter^89^. Although KLF15 alone has a modest effect on UCP1 expression, it significantly enhances UCP1 expression when acting together with KLF11^90^. Recent studies have revealed that KLF15 regulates the transcription of genes involved in lipid, glucose, and amino acid metabolism, thereby ensuring efficient fuel utilization for thermogenesis^91^, ^92^. In the absence of KLF15, the mice exhibit impaired nutrient oxidation, resulting in defective thermogenic function and an inability to maintain body temperature during fasting or cold exposure^93^. These findings provide important molecular insights into the development and functional regulation of brown adipocytes and may offer new therapeutic targets for obesity and related metabolic diseases.

### Other transcription factors

**Figure 6:**
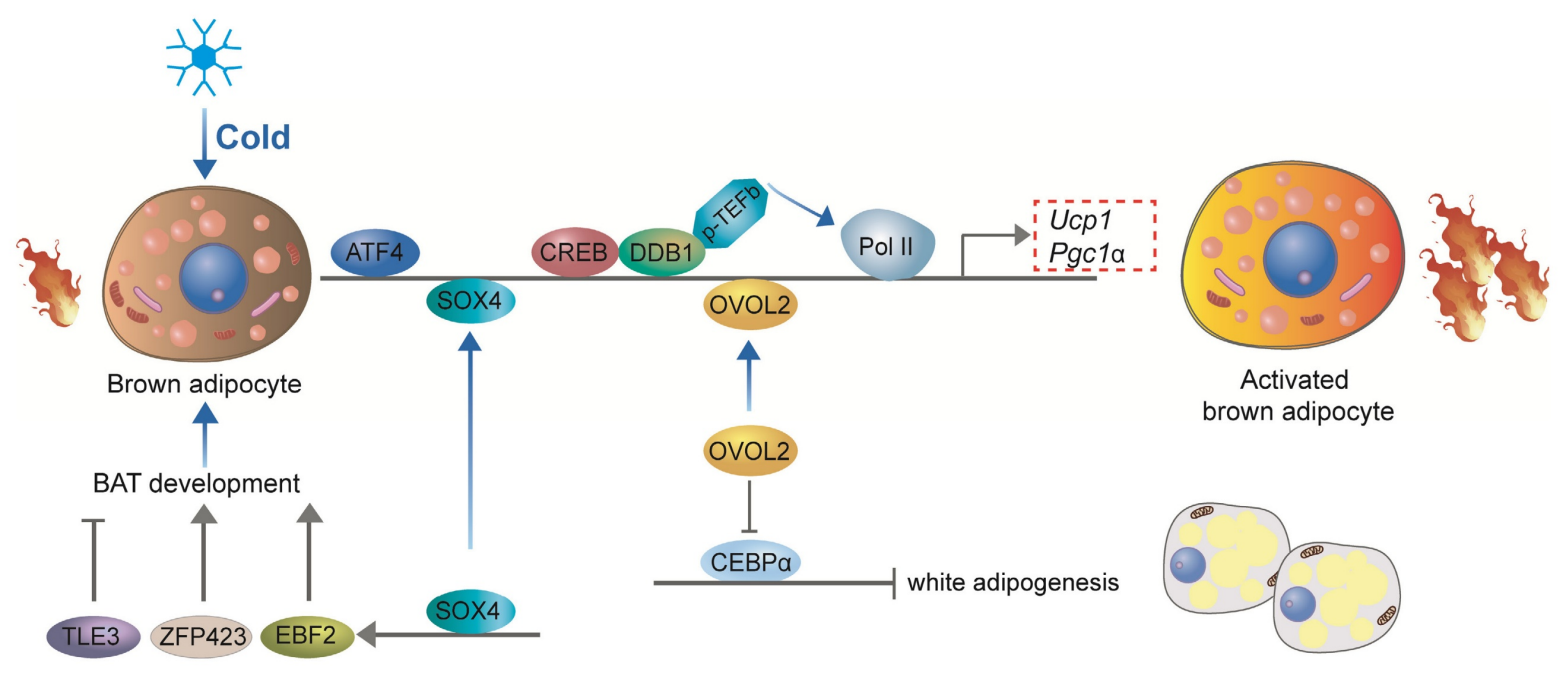
** Other transcriptional regulation of brown adipocyte development and thermogenesis**. DDB1 regulates thermogenic gene expression by recruiting P-TEFb, facilitating transcription in response to cold. ATF4 promotes *Ucp1* and *Pgc-1α* expression, enhancing thermogenesis and supporting amino acid metabolism. OVOL2 inhibits white adipocyte differentiation and supports thermogenesis. SOX4 activates EBF2 and thermogenic genes to promote BAT development. ZFP423 regulates adipogenesis and suppresses thermogenic gene expression in mature brown adipocytes. TLE3 inhibits thermogenesis by competing with PRDM16 and promoting a white adipocyte phenotype in BAT. These factors ensure brown adipocyte functionality for energy homeostasis and cold response.

Recent studies have underscored the pivotal roles of additional transcription factors in the development and thermogenic function of BAT. Damage-specific DNA binding protein 1 (DDB1) serves as a key regulator of thermogenic gene expression in brown adipocytes by binding to the promoters of critical thermogenic genes, including *Ucp1* and *Pgc-1α*. This interaction recruits the positive transcription elongation factor b (P-TEFb), which facilitates the release of paused RNA polymerase II (Pol II), enabling the rapid and coordinated transcription of these genes in response to acute cold exposure^94^. Activating Transcription Factor 4 (ATF4) is another essential factor for thermogenesis, as mice specifically deficient in ATF4 in BAT exhibit diminished thermogenic capacity and impaired cold tolerance. ATF4 promotes the expression of key thermogenic genes such as *Ucp1* and *Pgc-1α*, enhancing the thermogenic potential of BAT^95^. Furthermore, ATF4 regulates amino acid metabolism through activation of the mTOR signaling pathway, thereby further supporting thermogenic gene expression^96^, ^97^. Similarly, Ovo-like zinc finger 2 (OVOL2) is a critical regulator of both BAT development and thermogenesis. OVOL2-deficient mice display cold intolerance and reduced expression of thermogenic genes in BAT. OVOL2 interacts directly with the bZIP domain of C/EBPα, inhibiting its transcriptional activity and thus limiting the differentiation of white adipocytes^98^. SOX4 promotes BAT development by directly activating the transcription of EBF2. Additionally, SOX4 can directly bind to the promoter regions of thermogenic genes, such as *Prdm16* and *Ucp1*, thereby enhancing thermogenesis in mature brown adipocytes^53^. In ZFP423-deficient embryos, brown adipocyte differentiation is impaired, and BAT is significantly smaller and exhibits gross abnormalities compared to wild-type mice. Zfp423 directly regulates the expression of key adipogenic genes, as knockdown of Zfp423 in immortalized BAT precursor cells resulted in a decrease in PPARγ1 and PPARγ2 expression, which are crucial regulators of adipogenesis^99^. However, in thermogenic adipocytes, Zfp423 suppresses the thermogenic gene program by repressing the transcriptional activity of Ebf2^100^. The expression of transducin-like enhancer of split 3 (TLE3) protein in mice was significantly higher in WAT compared to BAT^101^. The overall gene expression profile of TLE3-expressing brown adipocytes was more reminiscent of white adipocytes, with decreased expression of several brown fat-selective markers, including *Dio2* and *Cldn1*^101^. In mature BAT brown adipocytes, TLE3 expression promotes a brown-to-white phenotypic switch^102^. TLE3 primarily inhibits the activity of Prdm16 by competing for binding sites on PPARγ, thereby suppressing the thermogenic gene program in brown adipocytes while simultaneously promoting the development and lipid storage of white adipocytes^102^. Together, DDB1, ATF4, OVOL2, SOX4, ZFP423, and TLE3 coordinate the development and thermogenic activity of brown adipocytes, ensuring their effective response to cold stimuli and their capacity to maintain body temperature and energy homeostasis (**Figure 6**).

## Major signaling pathways in thermogenesis and development of BAT

Single transcription factors alone cannot fully explain the mechanisms underlying brown adipose tissue development and thermogenesis. Exploring the crosstalk between signaling pathways is essential for understanding the development and thermogenic regulation of BAT.

The cAMP-PKA signaling pathway is a classical regulatory pathway for BAT thermogenesis. Norepinephrine (NE, Solarbio, Cat#N7960) released from the sympathetic nervous system activates β-adrenergic receptors (β-AR), which in turn activate adenylate cyclase (AC) to produce cAMP^103^, ^104^. PKA-induced activation of p38 MAPK leads to the phosphorylation of ATF-2, promoting PGC-1α and UCP1 transcription. Additionally, p38 MAPK directly phosphorylates and activates PGC-1α, which binds to PPARγ and the UCP1 promoter to enhance UCP1 expression^31^, ^34^, ^40^.

AMPK activation enhances glucose and fatty acid uptake, improves mitochondrial function, and promotes non-shivering thermogenesis of BAT^105^. AMPK deficiency impairs brown adipocyte differentiation and thermogenesis, leading to obesity and metabolic disorders^106^. NE increases PGC-1α expression similarly to AMPK activation, further enhancing thermogenic processes in BAT^107^, ^108^.

mTORC1 and mTORC2 are key regulators of BAT development and thermogenesis, although the exact mechanisms by which they exert their effects remain incompletely understood. mTORC1 is essential for BAT formation and thermogenic gene expression, promoting lipogenesis and mitochondrial function^109^, ^110^. However, in certain contexts, mTORC1 activation can suppress the expression of UCP1^111^, ^112^. Conversely, mTORC2 primarily governs glucose metabolism and lipid oxidation in BAT, with emerging evidence suggesting its role in thermogenesis via FoxO1 activation^113^-^115^. Despite progress, the interplay between mTORC1 and mTORC2 in thermogenic regulation remains unclear. Further research is needed to define the crosstalk between these complexes and their collective impact on thermogenesis.

Activation of Smad3 within TGF-β/BMP signalling pathway inhibits brown adipocyte differentiation and thermogenesis, primarily by suppressing key regulators such as PGC-1α^116^, ^117^. BMP4, while promoting beige adipocyte differentiation, reduces UCP1 expression in BAT^118^. In contrast, BMP7 (ABclonal, Cat#A0679) and BMP8a (LABLEAD) enhance BAT thermogenesis, with the thermogenic effect of BMP8a being estrogen-dependent in female mice^119^-^121^. Collectively, the TGF-β/BMP pathway tends to inhibit BAT differentiation and thermogenesis, although certain BMP family members exert context-dependent thermogenic effects^122^.

Several other signaling pathways also play crucial roles in regulating BAT development and thermogenesis. The cGMP-AKT pathway, activated by nitric oxide (NO) or natriuretic peptides (NPs), activates protein kinase G (PKG) and AKT, leading to enhanced mitochondrial biogenesis and the upregulation of thermogenic gene expression in BAT^123^, ^124^. In contrast, the Wnt/β-catenin signaling pathway negatively regulates BAT differentiation and thermogenesis by inhibiting adipogenesis and suppressing key transcription factors, such as PPARγ, C/EBPα, and PGC-1α^30^, ^125^, ^126^. LSD1 promotes brown fat formation by demethylating the H3K4 region within the wnt signaling module, thereby inhibiting wnt pathway activity^127^. The Notch signaling pathway facilitates thermogenesis by promoting thermogenic gene expression^128^. Furthermore, Hedgehog (Hh) signaling impedes brown preadipocyte differentiation and favors the differentiation of mesodermal precursors toward skeletal muscle, thus suppressing BAT formation^129^. Together, these pathways orchestrate a complex regulatory network that fine-tunes BAT development and thermogenesis.

## Challenges and opportunities in targeting BAT for obesity and metabolic disorders

Recent advancements in obesity pharmacotherapy have made notable strides in understanding mechanisms, enhancing efficacy, and ensuring safety, although certain limitations persist. Among these, GLP-1 receptor agonists, such as semaglutide (Nanjing Jiancheng) and liraglutide (GLPBIO, Cat#GC10311), effectively activate GLP-1 receptors in the hypothalamus, which in turn regulate appetite, delay gastric emptying, and increase satiety, leading to reduced food intake^130^, ^131^. Clinical trials have demonstrated weight loss ranging from 5% to 15%, although common gastrointestinal side effects, including nausea, vomiting, and diarrhea, have been observed^132^. On the other hand, SGLT2 inhibitors, such as empagliflozin (MCE, Cat#HY-15409), work by inhibiting renal glucose reabsorption, thereby promoting urinary glucose excretion and inducing caloric loss^133^, ^134^. These effects contribute to weight reduction and improved cardiovascular outcomes, though they may also increase the risk of certain adverse events.

Given that the safety of small-molecule drugs remains controversial, functional foods that promote BAT thermogenesis to combat obesity are receiving increasing attention. Capsaicin (Yeasen, Cat53616ES50) activates the transient receptor potential vanilloid 1 (TRPV1) receptor to increase energy expenditure and promote thermogenesis, thereby potentially benefiting weight regulation and combating obesity^135^. Camel milk-derived extracellular vesicles enhance BAT thermogenesis and improve metabolic health by activating the SOX4-EBF2 pathway^136^. Additionally, *Artemisia argyi* oil enhances BAT thermogenesis by directly activating ZFP516 expression and enhancing its interaction with LSD1^137^. As functional foods derived from natural sources, these options may present fewer side effects compared to synthetic drugs and could potentially serve as safe and effective dietary supplements in the fight against obesity in the future.

In recent years, significant progress in gene therapy, particularly through the use of AAV vectors, has shown considerable promise in overcoming some of the challenges associated with adipose tissue regulation^138^, ^139^. AAV vectors have proven to be a powerful tool for *in vivo* genetic manipulation of both WAT and BAT. Research has demonstrated that specific AAV serotypes, such as AAV8 and AAV9, can efficiently transduce adipocytes in adult mice, resulting in long-term and targeted transgene expression^5^, ^140^. This strategy has been applied to overexpress genes like hexokinase and vascular endothelial growth factor (VEGF) in adipose tissue, leading to enhanced glucose uptake and increased vascular density, respectively^141^. Wang *et al.* demonstrated that AAV-mediated overexpression of SOX4 (AAV-SOX4) significantly enhanced thermogenesis in BAT, resulting in increased heat production and reduced lipid accumulation. Furthermore, mice treated with AAV-SOX4 effectively mitigated obesity and metabolic disorders induced by a high-fat diet^53^. These findings highlight the potential of AAV-mediated genetic engineering as a therapeutic strategy for obesity and related metabolic disorders by directly targeting and modulating adipose tissue function.

The regulation of BAT and its thermogenic function is highly complex, involving a network of transcription factors/cofactors such as PGC-1α, EBF2, PRDM16, and PPARγ, which interact with various signaling pathways like cAMP-PKA and mTOR. These pathways exhibit context-dependent effects and are influenced by both cell-autonomous and non-cell-autonomous mechanisms, making it difficult to pinpoint precise therapeutic targets. Future research should focus on identifying key regulators of BAT thermogenesis, clarifying the complex interactions between signaling pathways, and exploring multi-target approaches. Additionally, examining age-related declines in BAT activity and developing more reliable animal models could provide valuable insights into activating human thermogenesis. The use of AAV vectors and other gene therapy techniques offers a promising avenue for targeted interventions that could enhance BAT thermogenesis and improve metabolic health^142^. Ultimately, these advancements could lead to new treatments for obesity and metabolic disorders by harnessing the therapeutic potential of BAT.

## Conclusions

BAT plays a crucial role in regulating energy metabolism and combating obesity, with its development and thermogenic function being subject to complex regulation by transcription factors and signaling pathways^122^. This review provides an in-depth exploration of the complex regulatory mechanisms governing the development and thermogenic function of BAT, revealing the close coordination between transcription factors and signaling pathways. Transcription elements such as PRDM16, PPARγ, EBF2, ZFP516, and FOXP1 play central roles in the development and thermogenesis of BAT, while signaling pathways such as cAMP-PKA, AMPK, and mTOR fine-tune BAT gene expression and metabolic function through interactions with these transcription factors. For example, EBF2 not only cooperates with PPARγ to activate the expression of thermogenic genes but also maintains the molecular characteristics of BAT by regulating chromatin accessibility. ZFP516, in turn, forms complexes with factors like PRDM16 to drive the expression of BAT-specific genes and, under cold exposure, activates UCP1 transcription through the cAMP-CREB/ATF2 signaling pathway. These findings suggest that the interaction between transcription factors and signaling pathways is crucial for regulating BAT function.

Members of the SIRT family and Kruppel-like factors also play important roles in BAT metabolic regulation. SIRT1 promotes thermogenesis by deacetylating PPARγ, enhancing its interaction with PRDM16, while SIRT3 activates CREB phosphorylation and upregulates PGC-1α expression, thereby boosting mitochondrial function and thermogenesis. Kruppel-like factors such as KLF11 and KLF15 directly bind to the UCP1 promoter region, significantly enhancing its expression and promoting BAT thermogenic function. The regulatory mechanisms of these factors not only reveal the complexity of BAT metabolic regulation but also provide a theoretical basis for the development of new therapeutic targets.

Enhancing the development and functionality of BAT is a promising strategy for addressing obesity and related metabolic disorders^143^. Promoting thermogenic activity of BAT is linked to increased energy expenditure, improved insulin sensitivity, and diminished hepatic lipid accumulation, all contributing to weight gain resistance^144^. Clinical and preclinical comprehensive studies have confirmed the presence of metabolically active brown fat in both infants and adults^145^. Additionally, elevated BAT activity in humans correlates with body weight reduction^19^. We have identified key transcription factors essential for the development and maintenance of BAT. Additionally, using AAV technology for genetic modifications in adipose tissue offers a novel approach for creating innovative treatments for obesity-related metabolic disorders^53^. Recent evidence demonstrates that incorporating human brown-like adipocytes into cell-based therapies confers substantial therapeutic efficacy in murine models^146^. However, despite the immense potential shown in animal models, clinical applications still face numerous challenges. These include how to precisely target BAT tissue, avoid potential immune responses, and ensure the long-term stability of gene expression. Furthermore, the complexity of both cell-autonomous and non-cell-autonomous mechanisms of signaling pathways and transcription factors adds to the difficulty of pinpointing precise therapeutic targets.

Future research should focus on several key areas: first, a deeper understanding of the interactions between transcription factors and signaling pathways, particularly the molecular mechanisms that underpin the cooperation between factors like EBF2, ZFP516, and FOXP1 with cAMP-PKA, AMPK, and mTOR pathways, in order to elucidate their synergistic regulation of BAT development and thermogenesis. Second, given the complexity of the BAT regulatory network, developing combination therapies targeting multiple key factors or pathways may be an effective strategy for improving therapeutic outcomes. Lastly, establishing animal models that more closely mimic human physiological and pathological states is critical for validating potential therapeutic targets and evaluating the efficacy of gene therapies.

## Figures and Tables

**Table 1:**
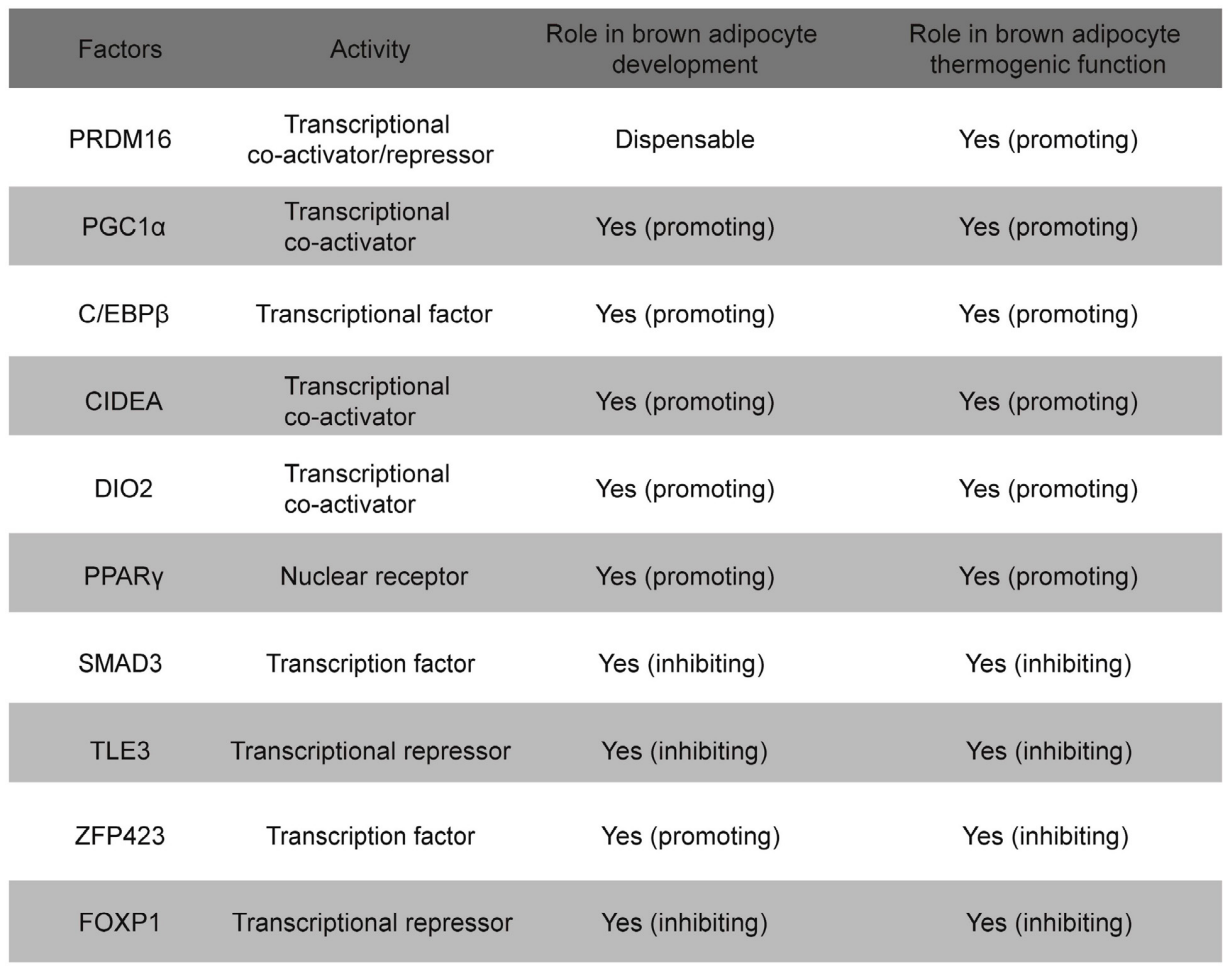
Major transcription factors and co-regulators in the regulation of BAT development and thermogenesis.

**Table 2:**
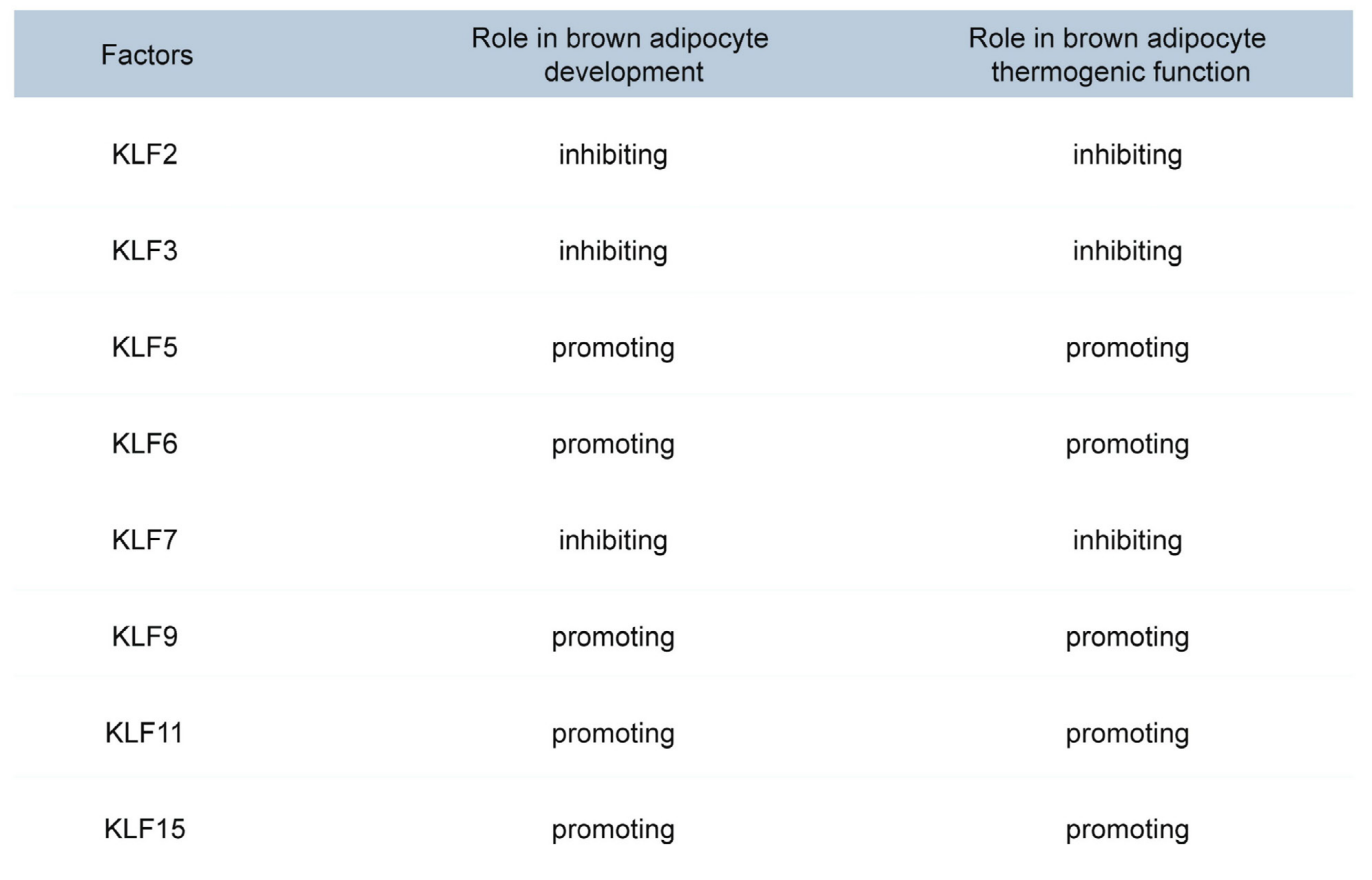
Roles of KLF family members in brown adipocyte differentiation and thermogenesis.

## Abbreviations

AMPK: AMP-activated protein kinase
ATF2: Activating transcription factor 2
ATF4: Activating transcription factor 4
BAT: Brown adipose tissue
BMP: Bone morphogenetic protein
cAMP-PKA: Cyclic AMP-protein kinase A
CREB: cAMP response element-binding protein
DDB1: Damage-specific DNA binding protein 1
EBF2: Early B cell factor 2
FOXP1: Forkhead box protein P1
LSD1: Lysine-specific demethylase 1
MAPK: Mitogen-activated protein kinase
mTOR: Mammalian target of rapamycin
NE: Norepinephrine
NP: Natriuretic peptide
OVOL2: Ovo-like zinc finger 2
PGC-1α: Peroxisome proliferator-activated receptor gamma coactivator 1α
PPARγ: Peroxisome proliferator-activated receptor gamma
PRDM16: PR domain containing 16
SOX4: SRY-related High Mobility Group box transcription factor 4
TGF-β: Transforming growth factor beta
TLE3: Transducin-like enhancer of split 3
UCP1: Uncoupling protein 1
WAT: White adipose tissue
ZFP423: Zinc finger protein 423
ZFP516: Zinc finger protein 516
